# Modulation of neuroinflammation by natural plant compounds: a promising approach for ischemic stroke

**DOI:** 10.3389/fphar.2025.1603417

**Published:** 2025-07-18

**Authors:** Pingping Niu, Yonggang Zhang, Binghao Wang, Sheng Qiu, Quanming Dong, Liqin Li, Yuntao Li

**Affiliations:** ^1^ Department of Neurosurgery, Huzhou Central Hospital, Fifth School of Clinical Medicine of Zhejiang Chinese Medical University, Huzhou, China; ^2^ Scientific Research Department, Huzhou Central Hospital, Fifth School of Clinical Medicine of Zhejiang Chinese Medical University, Huzhou, China; ^3^ Scientific Research Department, Zhejiang Chinese Medical University, Hangzhou, China

**Keywords:** ischemic stroke, natural plant compounds, neuroinflammation, neuroprotective, anti-inflammation

## Abstract

Ischemic stroke remains a leading global cause of mortality and long-term disability, imposing substantial health and economic burdens on society. Although interventions such as intravenous thrombolysis and mechanical thrombectomy have proven effective, their narrow therapeutic time window restricts accessibility for many patients. Post-ischemic brain injury is significantly driven by a cascade involving inflammatory cells and mediators, culminating in an “inflammatory storm” that damages neuronal cells. Consequently, targeting neuroinflammation following ischemic stroke to explore potential therapeutic strategies is of paramount importance. Natural plant compounds, bioactive constituents derived from plants, demonstrate considerable promise for ischemic stroke treatment, with many exhibiting potent neuroinflammation-modulating activities. This review synthesizes current research on natural plant compounds targeting post-ischemic stroke neuroinflammation and elucidates their underlying mechanisms of action. It aims to offer insights for developing plant-derived therapeutics specifically targeting neuroinflammation after ischemic stroke.

## 1 Introduction

Ischemic stroke remains a global health challenge characterized by disproportionately high rates of disability and mortality, posing significant threats to patient survival and quality of life. Although medical advancements have expanded therapeutic options, early pharmacological thrombolysis and mechanical thrombectomy persist as the primary evidence-based interventions. However, the narrow therapeutic time window severely limits clinical accessibility, with only a limited proportion of patients deriving benefit from these time-sensitive treatments (Feigin et al., 2025; Potter et al., 2022; Hilkens et al., 2024). This underscores the critical need to elucidate the pathophysiological mechanisms underlying post-ischemic neuronal injury and develop novel therapeutic strategies.

Neuroinflammatory cascades constitute a pivotal mechanism exacerbating cerebral tissue damage following ischemic stroke (DeLong et al., 2022). The ischemic insult triggers substantial production and release of inflammatory mediators, which initiate tissue injury and recruit peripheral immune cells. Subsequent immune cell infiltration establishes a self-perpetuating cycle: infiltrating leukocytes secrete additional proinflammatory cytokines that amplify tissue destruction while promoting further immune cell recruitment, culminating in a cytokine storm (Jayaraj et al., 2019; Wang H. et al., 2023; Zietz et al., 2024). Furthermore, inflammatory processes compromise blood-brain barrier integrity, facilitating cerebral edema formation and perpetuating neuroinflammation through enhanced peripheral immune cell trafficking (Chen et al., 2021). These mechanisms collectively position anti-inflammatory interventions as promising therapeutic targets for improving neurological outcomes in ischemic stroke.

Naturally derived compounds demonstrate considerable therapeutic potential in cerebral ischemia management (Ahsan et al., 2021). Current evidence indicates that specific botanical metabolites exhibit multi-target pharmacological activities: Gastrodia elata polysaccharides mitigate oxidative stress and ferroptosis pathways (Zhang Y. et al., 2024); Ginkgolide K modulates oxidative stress, neuroinflammation, and autophagy while promoting angiogenesis (Feng et al., 2019); Salidroside demonstrates dual anti-inflammatory and antioxidant properties (Fan et al., 2020). These natural plant compounds collectively target key pathological processes in ischemic stroke, including inflammation regulation, oxidative stress mitigation, ferroptosis inhibition, angiogenesis promotion, and autophagy modulation, thereby representing promising candidates for drug development.

This comprehensive review systematically evaluates the anti-inflammatory efficacy of naturally derived compounds in ischemic stroke, critically appraising current experimental evidence to inform the development of phytochemical-based therapeutics for post-stroke neuroinflammation management.

## 2 Literature search strategy

A literature search was conducted using the Web of Science and PubMed databases. The search covered the period from January 2005 to January 2025 to encompass research progress on natural plant compounds modulating neuroinflammation in ischemic stroke, including *in vivo*, *in vitro*, and clinical studies. We utilized a combination of Medical Subject Headings (MeSH) terms and free-text keywords, connected with Boolean operators, such as (“Ischemic Stroke” OR “Cerebral Ischemic “OR “Cerebral Ischemia-Reperfusion Injury”) AND (“Inflammation” OR “Neuroinflammation”) AND (“Natural plant compounds” OR “Natural Drugs” OR “Natural Medicine” OR “Herbal medicine” OR “Chinese Medicine”). Only peer-reviewed English articles were included, excluding case reports, editorials, and conference abstracts. The screening process involved an initial title and abstract review by two independent reviewers, with exclusions based on poor quality, outdated data, or lack of originality. Full texts of potentially eligible studies were then assessed. Any discrepancies arising during screening were resolved through discussion with a third reviewer or consensus.

## 3 Neuroinflammation in ischemic stroke

Acute cerebral ischemia is caused by reduced or obstructed blood flow, typically attributable to cardioembolism, small vessel occlusion, or atherosclerosis (Zhao et al., 2022). This impairment disrupts energy metabolism and initiates multiple pathophysiological processes, including secondary inflammatory responses. The neuroinflammatory mechanisms following ischemic stroke involve intricate interactions across several dimensions: (1) Glial cell activation and polarization post-ischemia: Microglia, the predominant immune cells in brain tissue, recognize damage-associated molecular patterns (DAMPs) through pattern recognition receptors (e.g., Toll-like receptor 4, TLR4), initiating M1 pro-inflammatory polarization and releasing substantial inflammatory mediators. Concurrently, ischemia induces astrocyte transformation into A1-type pro-inflammatory phenotypes, upregulating complement C3 expression to establish a neurotoxic microenvironment (Li X. et al., 2022). (2) Pro-inflammatory signaling activation: The NF-κB signaling cascade becomes activated following cerebral ischemia, characterized by IKK complex phosphorylation and subsequent IκBα degradation. This facilitates NF-κB nuclear translocation, driving transcriptional activation of COX-2, iNOS, and cytokine genes, thereby amplifying inflammatory responses and promoting blood-brain barrier disruption (Xu et al., 2024). (3) NLRP3 inflammasome assembly: Post-ischemic reactive oxygen species (ROS) accumulation and potassium efflux activate NLRP3, which recruits pro-caspase-1 via ASC adaptor proteins. This catalytic process facilitates IL-1β/IL-18 precursor maturation, inducing pyroptosis and promoting peripheral immune cell infiltration (Yang K. et al., 2024; Han et al., 2023). (4) Chemokine network regulation: Ischemic injury stimulates cerebral chemokine production and release, mediating peripheral immune cell recruitment. Specifically, the CXCL8/CXCR2 axis facilitates neutrophil transendothelial migration, while the CCL2/CCR2 pathway drives monocyte aggregation in injured areas, establishing a pro-inflammatory feedback loop between immune cells and inflammatory mediators (Wang et al., 2022). (5) Complement cascade activation: Post-ischemic C3a/C5a activation through G protein-coupled receptors upregulates endothelial adhesion molecules (ICAM-1/VCAM-1), promoting leukocyte rolling and extravasation. Simultaneously, C5b-9 membrane attack complexes directly induce neuronal membrane lysis (Széplaki et al., 2009; Si et al., 2019).

Collectively, the mechanisms underlying neuroinflammatory exacerbation of cerebral injury in ischemic stroke involve complex interactions. Ischemic tissue-derived inflammatory mediators directly damage neural cells while recruiting and activating immune cells. These activated immune cells not only inflict direct neuronal injury but also generate additional inflammatory mediators, establishing a self-perpetuating inflammatory cascade that amplifies cerebral tissue (Zietz et al., 2024).

## 4 Natural plant compounds alleviates neuroinflammation in ischemic stroke

**FIGURE 1:**
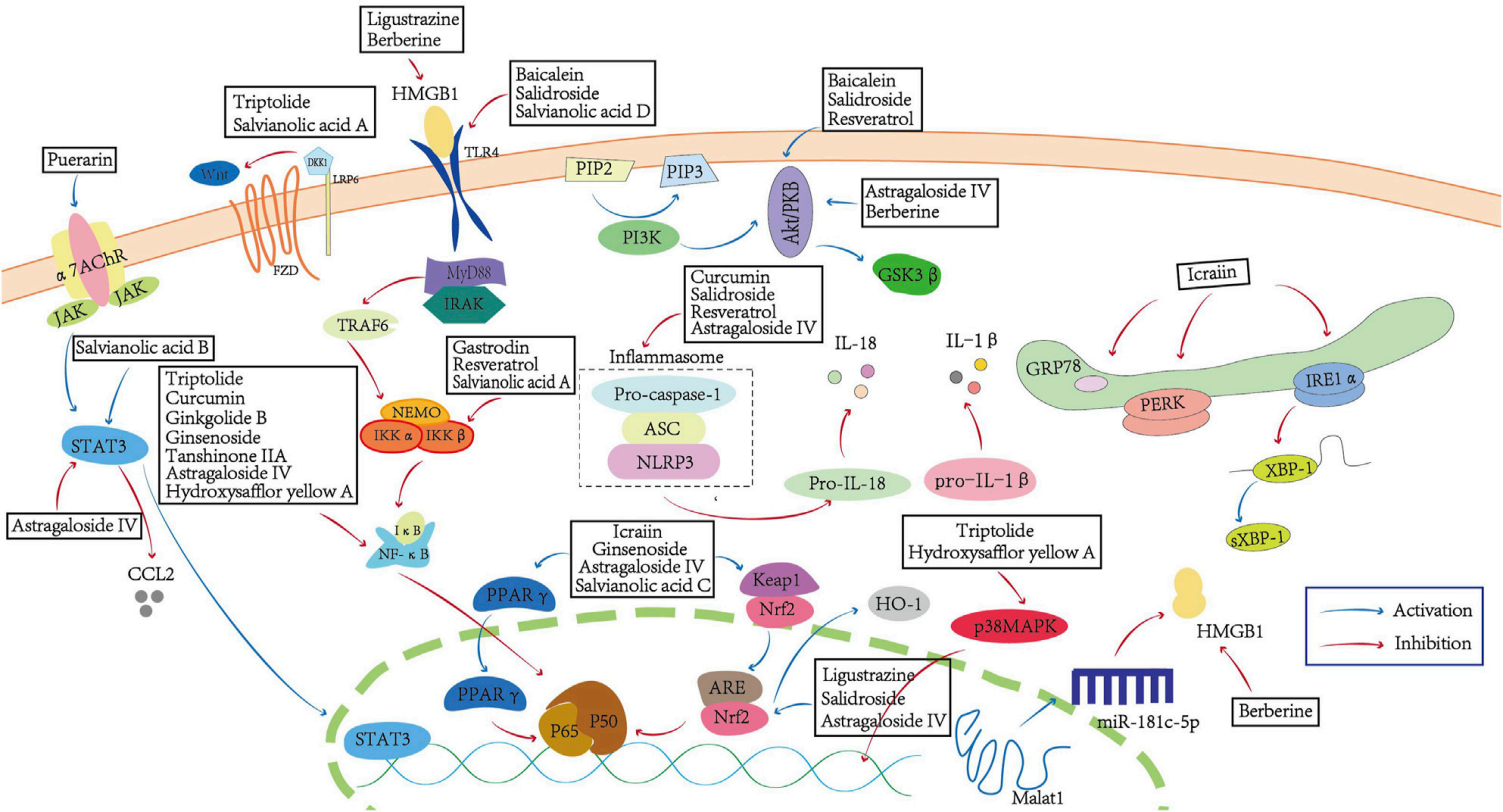
Natural plant compounds inhibit neuroinflammation in ischemic stroke. Natural plant compounds inhibit the release of inflammatory mediators and the activation of pro-inflammatory cells through multiple pathways such as the NF-κB signaling pathway, Wnt/β-catenin pathway, STAT3/VEGF signaling pathway, PI3K/AKT signaling pathway, and JAK/STAT signaling pathway.

The exploration of natural plant compounds has become a prominent area of interest in the ongoing search for innovative stroke treatments, offering considerable potential. Natural plant compounds exhibit a diverse array of bioactive properties in ischemic stroke therapy, encompassing autophagy regulation, ferroptosis inhibition, anti-inflammatory and immunomodulatory effects, oxidative stress mitigation, anti-apoptotic activity on neurons, blood-brain barrier preservation, and facilitation of neurovascular remodeling (Tao et al., 2020). Given the inherent complexity of traditional Chinese medicinal metabolites that exert multi-target mechanisms through diverse pathways, our review focuses on bioactive natural plant compounds relevant to the pathophysiological mechanisms of ischemic stroke, with particular emphasis on identifying potential therapeutic targets within neuroinflammatory responses. In this review, we collected and summarized the relevant research reports on five categories with anti-inflammatory activities after ischemic stroke. These natural plant compounds can exert anti-inflammatory effects through multiple mechanisms (Figure 1), including inhibiting the production of inflammatory mediators, the activation of glial cells, the activation of proinflammatory signals, and the infiltration of peripheral immune cells.

### 4.1 Polyphenols

Polyphenols with anti-inflammatory activity in ischemic stroke include salvianolic acid, gastrodin, curcumin, and resveratrol (Table 1).

**TABLE 1:** Polyphenols inhibit neuroinflammation in ischemic stroke.

Natural plant compound	PubChem CID	Sources	Model	Dose	Mechanisms	References
Salvianolic acid A	10364	*Salvia miltiorrhiza* Bunge [Lamiaceae]	OGD/R Rats MCAO model	62.5, 125, 250 μg/mL 5, 10 mg/kg	TLR2/4 signaling pathway	(Ling et al., 2021)
			MCAO model	50, 100 μg/kg	NF-κB signaling pathway	(Chien et al., 2016)
			Rats MCAO	5, 10, 20 mg/kg	MMP-9↓,NF-κB p65 activation↓	(Zhang et al., 2018)
Salvianolic acid B	6451084		Rats tMCAO	3, 6, 12 mg/kg	TLR4/MyD88/TRAF6 signaling pathway	(Wang et al., 2016)
			Rats MCAO model	10, 20, 40, 60 mg/kg	SIRT1 signaling	(Lv et al., 2015)
Salvianolic acid C	13991590		Rats tMCAO model	25 mg/kg	TLR4-TREM1-NF-κB pathway	(Guo et al., 2024)
			Rats tMCAO	20, 40 mg/kg	The M1 polarization of BV2 cell↓ Glycolysis↓ VEGFR2 and Notch1↑	(Shen et al., 2022)
Salvianolic acid D	75412558		PC12 cell OGD/R Rats MCAO/R model	1, 3, 15 mg/kg 0.2, 1,5 µM	HMGB1/NF-κB signaling pathway	(Zhang et al., 2020)
Gastrodin	115067	Gastrodia elata Blume [Orchidaceae]	OGD/R Rats MCAO/R mode	25, 50, 100 mg/kg 0.5, 5, 20 µM	JAK2/STAT3 signaling pathway	(Zhang et al., 2024b)
			Rats MCAO	100 mg/kg	CRP, IL-1β↓ Bcl-2, VEGF↑	(Wang et al., 2018a)
Curcumin	969516	Zingiberaceae Araceae	Rats MCAO	10 μmol/L	MAPK and NF-κB signaling pathways	(Lan et al., 2024)
			Rats dMCAO model	150 mg/kg 12.5.25 mmol/L	CD16, TNF-α, IL-12p70, IL-6↓ CD20↑	(Lei et al., 2023)
			Rats MCAO model	100 mg/kg	Ca^2+^ activation channel↓ P2X7R↓	(Liu et al., 2017)
Resveratrol	445154	*Vitis vinifera* L. [Vitaceae], Reynoutria japonica Houtt [Polygonaceae]	Rats MCAO/R	200 mg/kg	Th1/Th2 balance and Treg/Th17 balance	(Chiang et al., 2022)

OGD/R, Oxygen-glucose deprivation/reoxygenation; MCAO, Middle cerebral artery occlusion; I/R, ischemia reperfusion; MCAO/R, middle cerebral artery occlusion/reperfusion; OGD, oxygen-glucose deprivation; dMCAO, distal middle cerebral artery occlusion; BMECs, brain microvascular endothelial cells; ↓, downregulation or inhibition; ↑, upregulation or activation.

Salvianolic acid, derived from *Salvia miltiorrhiza* Bunge [Lamiaceae], is obtained from the dried roots and rhizomes of this medicinal plant. The phytochemical metabolites of *Salvia miltiorrhiza* Bunge are primarily divided into two groups: hydrophilic phenolic acids and lipophilic diterpenoids (Wang X. et al., 2020). Among these, salvianolic acid represents the predominant water-soluble fraction, comprising four bioactive metabolites: salvianolic acid A (SAA), salvianolic acid B (SAB), salvianolic acid C (SAC), and salvianolic acid D (SAD) (Zhao et al., 2024). SAA, SAB, SAC and SAD all show anti-inflammatory activity in ischemic stroke.

#### 4.1.1 Salvianolic acid A

Although SAA exhibits poor bioavailability in animal models, its concentration significantly increases in post-ischemic brain tissue, likely due to ischemia-induced disruption of the blood-brain barrier (Feng et al., 2017). Furthermore, the anti-inflammatory properties of SAA in post-ischemic stroke have been well established (Yang H. et al., 2024). Experimental evidence demonstrates that in ischemic stroke models, SAA administration not only significantly reduces the expression of pro-inflammatory cytokines IL-1β and TNF-α, but also effectively suppresses the activation of key inflammatory signaling pathways including TLR2/TLR4, MyD88, and NF-κB (Ling et al., 2021; Chien et al., 2016; Zhang et al., 2018). Furthermore, SAA treatment exhibits protective effects on blood-brain barrier (BBB) integrity, which may contribute to reducing peripheral immune cell infiltration (Chien et al., 2016). However, a notable limitation lies in the insufficient mechanistic depth of current investigations. Although Yun et al. observed attenuated SAA efficacy following TLR2/TLR4 intervention, these findings lack validation through *in vivo* experimental approaches. Moreover, critical rescue experiments remain necessary to conclusively establish TLR2/TLR4 as the pivotal molecular targets mediating SAA’s anti-inflammatory actions.

#### 4.1.2 Salvianolic acid B

SAB demonstrates significant anti-inflammatory properties in both cellular and animal models of ischemic stroke. *In vivo* studies revealed that SAB administration dose-dependently suppressed the expression of pro-inflammatory mediators (IL-1β, IL-6, IL-8, and MCP-1) within the ischemic penumbra. Moreover, it effectively attenuated the pathological overactivation of astrocytes and microglia following cerebral ischemia. *In vitro*, excessive activation of astrocytes and microglia after ischemia (Xu et al., 2017; Wang et al., 2016; Fan et al., 2018). *In vitro*, SAB can not only reduce OGD/R-induced neuronal cell damage, but also inhibit TLR4, MyD88, and NF-kB signals in neurons, and reduce the expression of proinflammatory factors IL-1β, IL-6, and TNF-α (Wang et al., 2016). Although the precise molecular targets of SAB remain to be fully elucidated, recent investigations by Hongdi Lv et al. have identified silent information regulator 1 (SIRT1) as a crucial mediator in SAB’s neuroprotective and anti-inflammatory mechanisms (Lv et al., 2015). However, the poor brain availability of SAB, evidenced by its minimal penetration into brain tissue following systemic absorption, remains a significant challenge (Zhang et al., 2021). Studies have demonstrated that SAB co-administered with borneol synergistically enhances its brain delivery (Zhang et al., 2021), thus offering a potential strategy to overcome its limited brain bioavailability.

#### 4.1.3 Salvianolic acid C

Although the therapeutic potential of SAC in ischemic stroke remains relatively under-investigated, emerging evidence highlights its anti-inflammatory properties. Following ischemic stroke, SAC exerts dual regulatory effects by suppressing both the activation of nuclear factor-κB (NF-κB) through inhibition of Toll-like receptor 4 (TLR4)/triggering receptor expressed on myeloid cells-1 (TREM1) signaling pathways in myeloid cells and microglial overactivation, thereby reducing the expression of proinflammatory mediators (Guo et al., 2024; Shen et al., 2022). Transcriptomic profiling further corroborates these findings, revealing SAC’s predominant modulation of neuroinflammatory pathways, acute-phase response signaling, and granulocyte adhesion processes, collectively underscoring its anti-inflammatory role in cerebral ischemia (Guo et al., 2024). Notably, molecular docking analyses by Yang et al. identified nine potential SAC-binding targets, among which peroxisome proliferator-activated receptor gamma (PPARγ) emerges as a critical therapeutic target (Yang et al., 2021). As a nuclear transcription factor abundantly expressed in macrophages and microglia, PPARγ orchestrates multifaceted neuroprotective mechanisms, including cerebral injury repair, anti-inflammatory response potentiation, and mitochondrial biogenesis. Intriguingly, SAC treatment upregulated PPARγ expression, suggesting its potential involvement in SAC-mediated anti-inflammatory effects (Yang et al., 2021; Titus et al., 2024). However, low bioavailability remains a challenge to be addressed for SAC (Song et al., 2016), and its pharmacokinetic profile in brain tissue also requires further elucidation.

#### 4.1.4 Salvianolic acid D

A study by Wen Zhang et al. investigated the therapeutic potential of salvianolic acid D (SAD) in ischemic stroke. Experimental evidence from both middle cerebral artery occlusion (MCAO) rat models and oxygen-glucose deprivation/reoxygenation (OGD/R)-induced cellular models demonstrated that SAD exhibited potent anti-inflammatory properties, as evidenced by its significant suppression of proinflammatory cytokines including TNF-α, IL-1β, and IL-6. Mechanistically, the anti-neuroinflammatory effects of SAD in ischemic stroke may be attributed to its inhibitory action on high mobility group box 1 (HMGB1) nuclear-to-cytoplasmic translocation and subsequent blockade of the TLR4/MyD88/NF-κB signaling cascade activation (Zhang et al., 2020). However, this hypothesis requires further experimental validation through comprehensive mechanistic studies. Besides, the direct molecular targets of SAD and its precise mode of action in modulating these pathways remain to be fully elucidated. Additionally, the pharmacokinetic characteristics of SAD in brain tissue also need to be explored.

#### 4.1.5 Gastrodin

Gastrodin is derived from the dried tuber of the orchid plant Gastrodia elata Blume [Orchidaceae], which has the effect of dispelling wind and unblocking circulation tracts. It has been used to treat central nervous system diseases such as dizziness, headache and epilepsy for hundreds of years (Xiao et al., 2023). Gastrodin can be rapidly absorbed in the intestine and widely distributed in the body, and can pass through the BBB into the brain tissue (Dai et al., 2024). Gastrodin exhibits potent anti-inflammatory properties in various disease models by modulating oxidative stress and the TLR4/NF-κB signaling pathway. In ischemic tissue, endogenous DNA damage mediated by TLR9 exacerbates post-ischemic inflammatory responses, which can be effectively suppressed by gastrodin through downregulation of TLR9 expression. Furthermore, gastrodin may directly bind to JAK2 and reduce the production of inflammatory cytokines after cerebral ischemia by inhibiting JAK2/STAT3 signaling. In addition, as oxidative stress represents a critical factor in exacerbating neuroinflammation, gastrodin also demonstrates regulatory effects on post-ischemic oxidative stress (Zhang M. et al., 2024; Wang S. et al., 2018; Peng et al., 2015). Notably, a clinical study demonstrated that Gastrodin effectively prevented postoperative delirium following cardiac surgery, with no drug-related adverse events reported, highlighting its favorable safety profile in clinical settings (Bai et al., 2025). These findings provide critical support for advancing future clinical trials investigating the therapeutic potential of Gastrodin in ischemic stroke management.

#### 4.1.6 Curcumin

Curcumin is a natural plant compound extracted from the roots and stems of some plants of Zingiberaceae and Araceae, which has anti-inflammatory and antioxidant effects. NOD-like receptor protein 3 (NLRP3) inflammasome mediated microglial pyroptosis is a pro-inflammatory programmed cell death, which plays a crucial role in neuroinflammation and functional recovery after stroke (Han et al., 2023). NF-κB signal transduction is the main pathway to promote inflammatory response, and plays a central role in the aggregation of NLRP3 metabolites and the activation of NLRP3 inflammasome (Lei et al., 2023). Curcumin can downregulate MAPK and NF-κB signal transduction pathway, inhibit the activation of NLPR3 inflammasome, reduce the expression of ICAM-1, MMP-9 and IL-1β exerting anti-inflammatory effect (Yang X. et al., 2024; Wang et al., 2024). Curcumin can also act on the activation stage of microglia, inhibit M1 polarization, promote M2 polarization, inhibit Ca^2+^ activation channel and P2X7 receptor (P2X7R) to inhibit inflammatory reaction (Bissacotti et al., 2023; Lan et al., 2024; Liu et al., 2017; Wang Z. et al., 2020). Notably, poor water solubility, low oral bioavailability, and poor BBB permeability have limited the application of curcumin in the past, but the application of nanotechnology has effectively improved this situation. Currently, nanoparticles loaded with curcumin have been used in the study of ischemic stroke and have shown excellent anti-inflammatory effects (Yang Q. et al., 2024; Li F. et al., 2021). Curcumin is a natural plant compound with great potential for the treatment of ischemic stroke. However, curcumin has been identified as a pan-assay interference compound (Baker, 2017), which requires researchers to focus on in related studies.

#### 4.1.7 Resveratrol

Resveratrol is a stilbene metabolite famously found in *Vitis vinifera* L. [Vitaceae] and Reynoutria japonica Houtt [Polygonaceae] (Fletes-Vargas et al., 2023). It has been widely studied for its cardioprotective and neuroprotective effects. In a cerebral ischemia model, resveratrol exhibits anti-inflammatory and antioxidant effects. Specifically, the anti-inflammatory mechanisms of resveratrol include inhibiting the activation of NF-κB and NLRP3 inflammasomes to reduce the production of inflammatory factors, downregulating the cluster of differentiation 147 (CD147)/MMP-9 axis to inhibit microglial activation and the release of inflammatory factors, activating the PI3K/AKT signaling pathway to participate in the anti-inflammatory process, and regulating the changes in Th17/Tregs and Th1/Th2 polarity mediated by the intestinal lamina propria to inhibit systemic inflammation and neurological symptoms after stroke (Tsai et al., 2022; Chiang et al., 2022; Dou et al., 2019). Furthermore, A systematic review evaluated the research on resveratrol in animal models of ischemic stroke and pointed out that resveratrol has beneficial effects on rodent stroke models (López-Morales et al., 2023), suggesting that resveratrol has great potential for the treatment of ischemic stroke. Resveratrol exhibits a short biological half-life, poor water solubility at physiological pH, and low absorption rate, which limits its clinical application. However, nanostructure-based drug delivery systems have shown significant potential to enhance the chemical stability, water dispersibility, bioavailability, and blood-brain barrier (BBB) permeability of resveratrol (Ramalingam and Ko, 2016). Besides, as a pan-assay interference compound (Baell and Walters, 2014), Resveratrol also needs to be paid special attention in research.

### 4.2 Flavonoids

Flavonoids with anti-inflammatory activity in ischemic stroke include Baicalein, Icraiin, Puerarin, and Hydroxysafflor yellow A (Table 2).

**TABLE 2:** Flavonoids inhibit neuroinflammation in ischemic stroke.

Natural plant compound	PubChem CID	Sources	Model	Dose	Mechanisms	References
Baicalein	64982	*Scutellaria baicalensis* Georgi [Lamiaceae]	Neuron–astrocyte cocultures OGD/R model	137.5, 68.75, 34.38, 17.19, 8.59, 4.30, 2.15, 1.08 μg/mL	BDNF-TrkB/PI3K/Akt and MAPK/Erk1/2 signaling axes	(Baell and Walters, 2014)
			BV2 and Raw 264.7 cell	0.5, 1, 5, 10, 50, 150, 200 μg/mL	PI3K/AKT/mTOR pathway	(Sowndhararajan et al., 2017)
			BMECs OGD/R model	5, 10, 25 μg/mL	INOS, IL6↓	(Yu et al., 2023)
Icariin	5318997	Epimedium brevicornu Maxim [Berberidaceae]	Microglia OGD/R model	0.37, 0.74, 1.48 μmol/L	IRE1/XBP1s pathway	(Seyedi et al., 2023)
			Rat MCAO model	60 mg/kg	PPARs/Nrf2/NF-κB and JAK2/STAT3/NF-κB pathways	(Mo et al., 2021)
Puerarin	5281807	*Pueraria montana* var. Lobata [Fabaceae]	Rats MCAO model	25, 50, 100 mg/kg	SIRT1/HIF-1α/VEGF signaling pathway	(Dai et al., 2021)
			Rats MCAOmodel	36, 54 mg/kg	α7AChR/JAK2/STAT3 signaling pathway NF-κBp65 protein, IL-1β, IL-6, TNF-α↓	(An et al., 2021)
Hydroxysafflor yellow A	6443665	*Carthamus tinctorius* L. [Asteraceae]	BV2 cell OGD/R model	20, 40, 80, 160, 320, 640, 1,280 µM	NF-κB and p38 MAPK pathway↓	(Yi et al., 2017)

OGD/R, Oxygen-glucose deprivation/reoxygenation; OGD, oxygen-glucose deprivation; I/R, ischemia reperfusion; MCAO, middle cerebral artery occlusion; TBI, Traumatic brain injuries; ↓, downregulation or inhibition; ↑, upregulation or activation.

#### 4.2.1 Baicalein

Baicalein, a flavonoid compound extracted from the roots of *Scutellaria baicalensis* Georgi [Lamiaceae], can cross the BBB and penetrate brain tissue within 20–30 min after administration (Sowndhararajan et al., 2017). It has anti-inflammatory and anti-thrombotic effects, and is commonly used in clinical treatment of stroke. It can reduce the increase of NO and MDA after oxygen-glucose deprivation/reoxygenation (OGD/R), reduce TNF-α, IL-1β and IL-6 expression, increase the expression of SOD, which can be reversed by tropomyosin receptor kinase B (TrkB) antagonist ANA-12, indicating that brain-derived neurotrophic factor (BDNF)-TrkB signal transduction is essential for its neuroprotective effect (Li C. et al., 2021). In addition to BDNF-TrkB signal transduction, baicalin also participates in the activation of PI3k/AKT signaling pathway and the inhibition of NF-κB signaling pathway (Long et al., 2025). PI3K/AKT signaling pathway is an important downstream cascade of BDNF-TrkB signaling transduction, and inhibition of inflammatory reaction is the direct consequence of PI3K/AKT signal transduction (Yu et al., 2023). The inhibition of NF-κB signaling pathway is targeted on the TLR upstream. By down-regulating the TLR, suppress the activation of NF-κB and block the signal from being sent to the downstream factor TNF-α, in order to resist immune cell infiltration and relieve ischemic stroke (Lu et al., 2025; Hao et al., 2023). Baicalin exerts anti-inflammatory effects in ischemic stroke through multiple pathways. Furthermore, baicalin-loaded nanoparticles delivered via the nose-to-brain route demonstrate significant efficacy in suppressing oxidative stress and neuroinflammation in rat models of ischemic stroke, indicating promising therapeutic potential (Li X. et al., 2022).

#### 4.2.2 Icariin

Icariin, one of the main active metabolites of Epimedium brevicornu Maxim [Berberidaceae] has many effects, such as enhancing blood flow, regulating immune function, and enhancing bone growth (Seyedi et al., 2023). It was found that icariin could directly regulate glucose-regulated protein 78 (GRP78), inositol requiring enzyme-1α (IRE1α) and protein kinase RNA-like ER kinase (PERK) to inhibit endoplasmic reticulum (ER) stress and repress the inositol-requiring enzyme 1 (IRE1)/X-box binding protein 1 (XBP1s) pathway, reduce microglial activation in the ischemic penumbra, decrease the expression and release of proteins such as IL-1β and TNF-α to exert anti-inflammatory effects to attenuate ischemic stroke injury (Zheng et al., 2022; Mo et al., 2021; Tao et al., 2024). PPARs are endogenous neuroprotective factors and have three different PPAR isoforms, PPARα, PPARβ and PPARγ. Activation of PPAR subtypes, especially PPARγ, was shown to protect against post-ischemic inflammation and neuronal damage (Titus et al., 2024). Cytokines and oxidative stress can stimulate the JAK phosphorylation, which in turn leads to STAT phosphorylation and is involved in the pathogenesis of inflammatory and autoimmune diseases. Icariin could regulate PPARs and repress JAK2/STAT3 pathway, which in turn inhibited NF-κB activation mediated inflammation to exert neuroprotective effects (Dai et al., 2021). Furthermore, studies demonstrate that icariin is extensively absorbed in the bloodstream and rapidly distributes to the brain within 15–20 min. Thus, icariin exhibits considerable potential for clinical treatment of ischemic stroke (An et al., 2021).

#### 4.2.3 Puerarin

Puerarin is a single metabolite flavonoid glycoside extracted from the dried roots of legume plant *Pueraria montana* var. Lobata [Fabaceae] that has effects of activating blood circulation and eliminating stasis, improving microcirculation, and dilating blood vessels. The neuroprotective effect of Puerarin against cerebral ischemic injury was manifested by attenuating hypoxia-inducible factor-1α (HIF-1α) and TNF-α expression, inhibiting inflammatory responses, apoptosis, and neutrophil activation (Liu et al., 2023). Puerarin pretreatment can inhibit NF-κB expression and by stimulating α7 nicotinic acetylcholine receptor (α7AChR) activates the JAK3/STAT3 signaling pathway to regulate inflammatory responses (Liu et al., 2013). However, the clinical application of puerarin is significantly limited by its suboptimal physicochemical properties and BBB permeability, evidenced by a low brain-to-serum area under the curve (AUC) ratio of only 2.92% following intravenous administration. Notably, research by Tao Yi et al. demonstrates that co-administration of borneol with a self-microemulsifying drug delivery system (SMEDDS) enhances the oral absorption and brain penetration of puerarin in mice (Yi et al., 2017), suggesting a promising strategy for developing oral puerarin formulations for ischemic stroke.

#### 4.2.4 Hydroxysafflor yellow A (HSYA)

Carthamus tinctorius L. [Asteraceae] has the efficacy of activating blood circulation, eliminating stasis and relieving pain, and HSYA is the most effective water-soluble site for the pharmacological efficacy of Carthamus tinctorius L. *In vitro*, cell experiments found that HSYA could reduce the levels of pro-inflammatory factors after OGD, inhibit the OGD-induced degradation of IκB and nuclear translocation of p65 and the phosphorylation of p38 MAPK, exerting anti-inflammatory properties, enhances cell viability, and inhibits cell death (Zhao et al., 2020; Li et al., 2013). *In vivo*, HSYA decreased the levels of inflammatory marker TNF-α and reduced neuroinflammation, which could be reversed by an opener of the mitochondrial permeability transition pore (mPTP), indicating that HSYA might exert neuroprotective effects by inhibiting the opening of the mPTP (Yi et al., 2022). Furthermore, despite its slow rate of absorption from blood into cerebrospinal fluid, HSYA can penetrate brain tissue and exert neuroprotective effects following ischemic stroke (Du et al., 2022; Sheng et al., 2020).

### 4.3 Saponins

Saponins with anti-inflammatory activity in ischemic stroke include Ginsenoside Rd, Astragaloside IV, and Salidroside (Table 3).

**TABLE 3:** Saponins inhibit neuroinflammation in ischemic stroke.

Natural plant compound	PubChem CID	Sources	Model	Dose	Mechanisms	References
Ginsenoide Rg1	441923	*Panax ginseng* C.A.Mey. [Araliaceae], *Panax quinquefolius* L. [Araliaceae], *Panax notoginseng* (Burkill) F.H.Chen [Araliaceae]	Rats MCAO model	20, 40 mg/kg	BDNF↑ IL-1β, IL-6, TNF-α, Glu,Asp↓	(Zhou et al., 2024)
			Rats I/R model	10, 20, 40 mg/kg	IκBα phosphorylation and NF-κB nuclear translocatin↓ Proteasomal activity and protein aggregate accumulation↓	(Mohebbati, 2023)
Ginsenoside Rb1	9898279		Rats MCAO/R model	50 mg/kg	PPARγ signaling pathway	(Wang et al., 2018b)
			Rats MCAO model	5, 20, 40 mg/kg	Nitric oxide synthase, IL-1β, MMP9, NOX↓ Arginase 1, IL-10↑ Tight junction proteins↑	(Zheng et al., 2019)
Astragaloside IV	13943297	*Astragalus mongholicus* (Fisch.) Bunge [Fabaceae]	OGD/R Rats MCAO/R model	10 mL/kg 5, 10, 20, 40, 80 μg/mL	NLRP3/Caspase-1/GSDMD pathway	(Li et al., 2021c)
			Rats I/R model	28, 56 mg/kg	NLRP3, NF-κB, Caspase-1, IL-1β, IL-18↓	(Chen et al., 2022)
			Rats MCAO	20.40 mg/kg	STAT3↓ NK cell infiltration and activation↓	(Tang et al., 2019)
			Photochemical brain ischemia model, IL-17 knock out mice	200 mg/kg	Akt/GSK-3β and Wnt/β-catenin pathway	(Hou et al., 2024)
Salidroside	159278	*Rhodiola rosea* L. [Crassulaceae]	BV2 cell OGD/R model, Rats MCAO/R model	6.25, 12.5, 25, 50, 100 µM 50 mg/kg	TLR4/NF-κB signaling pathway	(Sun et al., 2020)
			Rats MCAO/R model	50 mg/kg	PI3K/Akt/HIF Signaling pathway	(Stępnik and Kukula-Koch, 2020)
			Rats pMCAO model	25, 50, 100 mg/kg	PI3K/PKB/Nrf2/NFκB Signaling pathway	(Liu et al., 2021)

OGD, Oxygen-glucose deprivation; I/R, ischemia reperfusion; MCAO, middle cerebral artery occlusion; OGD/R, oxygen-glucose deprivation/reoxygenation; MCAO/R, middle cerebral artery occlusion/reperfusion; NSCs, neural stem cells; tMCAO, transient middle cerebral artery occlusion; pMCAO, permanent middle cerebral artery occlusion; ↓, downregulation or inhibition; ↑, upregulation or activation.

#### 4.3.1 Ginsenoside Rd

Ginsenosides are regarded as the main active metabolites of ginseng, which can be extracted from Araliaceae such as *Panax ginseng* C.A.Mey. [Araliaceae], *Panax quinquefolius* L. [Araliaceae] and *Panax notoginseng* (Burkill) F.H.Chen [Araliaceae], contain various monomeric saponin metabolites such as Rd, Rg1 and Rb1, are widely studied. The mechanism underlying the neuroprotective effect of Rd is the inhibition of microglial proteasome activity, thereby inhibiting microglial activation and reducing inflammatory factors, iNOS, and COX-2 expression, followed by the inhibition of inhibitory subunit of IκBα phosphorylation and NF-κB nuclear translocation mediated inflammation, and its anti-inflammatory inhibition had significantly less adverse effects than glucocorticoids (Zhou et al., 2024); Rg1 reversed cerebral I/R-induced increases in IL-1β, caspase-1, ASC, and NLPR1. Alleviates neuroinflammation in ischemic stroke by inhibiting NADPH oxidase 2 (NOX2) mediated ROS accumulation and Ca^2+^ mediated calcineurin-nuclear factor of activated t-cells 1(CN-NFAT1) activation (Mohebbati, 2023). Its anti-inflammatory mechanisms also include decreased pro-inflammatory factor expression, inhibition of IκBα phosphorylation and NF-κB nuclear translocation, decreases proteasome activity and protein aggregate accumulation in the brain (Wang L. et al., 2018; Zheng et al., 2019). RB1 can activate PPARγ, decrease phosphorylated NF-κB p65 levels, which inhibit the production of proinflammatory cytokines, as well as neuroinflammation by inhibiting MMP-9 and NADPH oxidase 4 (NOX4) derived free radicals, thereby maintaining the integrity of the BBB ischemic stroke and ameliorating brain injury (Su et al., 2022; Chen et al., 2015; Yu et al., 2024). Although ginsenosides exhibit the lowest distribution to the brain compared to other organs, their brain-to-serum ratio was significantly higher than that of albumin, indicating their ability to cross the BBB (Ye et al., 2013).

#### 4.3.2 Astragaloside IV

Astragaloside IV, the most major active metabolite of *Astragalus mongholicus* (Fisch.) Bunge [Fabaceae] membranaceus, has shown positive therapeutic effects on ischemic stroke in both animal and cellular experiments. Inflammation is one of the essential pathological processes in ischemic stroke, and the anti-inflammatory mechanisms of Astragaloside IV include activation of Nrf2 to inhibit NLRP3/caspase-1/Gasdermin-D (GSDMD) pathway mediated pyroptosis, inhibition of NLRP3 inflammasome activation and NF-κB phosphorylation, increased PPARγ expression, promotes the polarization of microglia/macrophages from M1 to M2 phenotype, enhances the secretion of neurotrophic and growth factors by M2 microglia/macrophages in the ischemic area, and promotes neurogenesis and angiogenesis (Li L. et al., 2021; Chen et al., 2022; Xiao et al., 2021; Tang et al., 2019; Hou et al., 2024). Inhibition of STAT2 activation to suppress CCL3 mediated brain infiltration of NK cells, reduced NK cell transcription of Natural Killer Group 2D and IFN-γ release, attenuated NK cell-mediated cytolytic killing of neurons and brain injury (Li S. et al., 2021). Downregulation of IL-7 protein expression, followed by upregulation of AKT/glycogen synthase kinase-3β (GSK-3β) and Wnt/β-catenin pathway, which activates the stemness of neural stem cells, inhibits neuronal apoptosis, promotes neurogenesis, and ultimately alleviates cognitive deficits after stroke (Sun et al., 2020). Although pharmacokinetic studies indicate that astragaloside IV exhibits poor oral bioavailability and blood-brain barrier (BBB) permeability, it can be detected in brain tissue following ischemic stroke (Du et al., 2022; Stępnik and Kukula-Koch, 2020). This phenomenon is likely attributable to BBB disruption after ischemic stroke.

#### 4.3.3 Salidroside

Salidroside, the main active metabolite of *Rhodiola rosea* L. [Crassulaceae], has anti-inflammatory, antioxidant, immunomodulatory, free radical scavenging, and inhibitory effects on neural apoptosis. The anti-inflammatory effects of salidroside involve multiple signaling pathways, including inhibition of TLR3/NF-κB signaling pathway that inhibits cerebral I/R-induced NLRP4 inflammasome activation and apoptosis in microglia. Activation of the PI3K/AKT/HIF signaling pathway reduces the expression of integrin alpha-M (CD11b) and inflammatory mediators. Regulation of PI3K/PKB/Nrf2/NF-κB signaling pathways to reduce neuroinflammation and nerve injury (Liu et al., 2021; Wei et al., 2017; Zhang et al., 2019). Moreover, salidroside can also promote microglial M2 polarization, enhance microglial phagocytosis and inhibit microglial derived inflammatory cytokine release (Ji et al., 2025). Despite demonstrating significant anti-inflammatory efficacy following ischemic stroke, salidroside exhibits extremely low bioavailability in the brain based on pharmacokinetic studies—a critical challenge requiring resolution (Fan et al., 2020).

### 4.4 Diterpenoids

Diterpenoids with anti-inflammatory activity in ischemic stroke include Tanshinone IIA, Triptolide, and Ginkgolide B (Table 4).

**TABLE 4:** Diterpenoids inhibits neuroinflammation in ischemic stroke.

Natural plant compound	PubChem CID	Sources	Model	Dose	Mechanisms	References
Tanshinne IIA	164676	*Salvia miltiorrhiza* Bunge [Lamiaceae], *Saxifraga stolonifera* Curtis [Saxifragaceae]	Rats MCAO model	8 mg/kg	GFAP, caspase-3, caspase-8↓	(Arefnezhad et al., 2024)
			Rats I/R model	4, 8 mg/kg	NeuN, Protein disulfide isomerase, Na, K-ATPase↑ Microglial activation↓	(Carpi et al., 2023)
			Rats pMCAO model	5, 10, 20 mg/kg	SOD↑ MDA, NO, iNOS, NF-κB nuclear translocatin↓	(Zhou et al., 2015)
			Rats I/R model	25 mg/kg	MIF, MPO, IL-6, TNF-α, NF-κB↓	(Wen et al., 2016)
Triptolide	107985	*Tripterygium wilfordii* Hook.f. [Celastraceae]	PBMCs and NK Cells	0.4, 2, 5 ng/mL	p38MAPK signaling pathways	(Long et al., 2020)
			Rats I/R model	12.5, 25, 50 mg/kg	Wnt/β-catenin signaling pathway SOD↑ MDA, ROS↓	(Gao et al., 2021)
Ginkgolide B	11973122	*Ginkgo biloba* L. [Ginkgoaceae]	BV2 microglia, macrophages, Rats tMCAO model	5 μg/mL 5 mg/kg	Microglia/macrophage M2 polarization↑	(Pan and Xu, 2020)
			Rats tMCAO model	10, 20, 40 mg/kg	NF-κB signaling pathway	(Yang et al., 2024e)

MCAO, Middle cerebral artery occlusion; I/R, ischemia reperfusion; pMCAO, permanent middle cerebral artery occlusion; OGD, oxygen-glucose deprivation; tMCAO, transient middle cerebral artery occlusion; BBB, blood-brain barrier; ↓, downregulation or inhibition; ↑, upregulation or activation.

#### 4.4.1 Tanshinone IIA

Tanshinone IIA can be extracted from *Salvia miltiorrhiza* Bunge, *Saxifraga stolonifera* Curtis [Saxifragaceae] and other traditional Chinese medicines, it has many effects such as improving coronary circulation, anti-atherosclerosis, and anti-inflammation. Cerebral I/R injury is closely related to the inflammatory response, and Tanshinone IIA can relieve the pro-inflammatory response induced by I/R. The mechanisms include reducing the expression of GFAP, inhibiting the excessive activation of astrocytes, inhibiting the activation of microglia, downregulating the expression of macrophage migration inhibitory factor in neurons, decreasing NF-κB activation and the release of various inflammatory factors, inhibiting oxidative stress and free radical mediated inflammatory damage (Xie et al., 2023; Arefnezhad et al., 2024; Carpi et al., 2023; Zhou et al., 2015; Wen et al., 2016; Dong et al., 2009; Chen et al., 2012). Studies indicate that Tanshinone IIA monomer exhibits poor BBB permeability; however, borneol significantly enhances its delivery to brain tissue (Zhang et al., 2021). Furthermore, intranasal administration demonstrates considerable potential in facilitating Tanshinone IIA transport to the brain (Long et al., 2020).

#### 4.4.2 Triptolide

Triptolide is an important diterpene active compound in *Tripterygium wilfordii* Hook.f. [Celastraceae] Since Kupchan et al. first isolated triptolide and found its significant anti-leukaemic effects, it has also been proven to have significant anti-inflammatory, immunosuppressive, anticancer and other important biological activities (Gao et al., 2021). Triptolide is a strong anti-inflammatory agent, reduces astrocyte activation, downregulates NF-κB and p38 MAPK signaling pathway, and inhibits the release of IL-1β, iNOS, COX-2, TNF-α and other proinflammatory factors, alleviates BBB damage (Wang N. et al., 2023). In addition to the classical NF-κB pathway the mechanism of Triptolide treatment in ischemic stroke also involves inhibition of inflammatory responses mediated by activation of the Wnt/β-catenin signaling pathway (Pan and Xu, 2020). Furthermore, its favorable BBB permeability and comparable pharmacokinetic profiles simultaneously observed in cerebral and systemic compartments highlight triptolide’s promise as a clinically translatable natural plant compound (Zhu et al., 2020).

#### 4.4.3 Ginkgolide B

Ginkgolides, a class of active metabolites extracted from *Ginkgo biloba* L. [Ginkgoaceae], of which Ginkgolide B is the most physiologically active and the strongest antagonist of platelet activating factor discovered to date, are commonly used in the clinic to treat ischemic stroke and also have protective effects on damaged neurons. The protective function exhibited by Ginkgolide B in ischemic stroke, such as reducing infarct volume, ameliorating neurological deficits, and alleviating the increase in BBB permeability, is associated with its anti-inflammatory properties. Both *in vivo* and *in vitro* experiments have confirmed its ability to promote the conversion of microglia/macrophages from an inflammatory M1 phenotype to an anti-inflammatory M2 phenotype (Li et al., 2023; Yang Y. et al., 2024); Ginkgolide B anti-inflammatory mechanism mainly involves NF-κB signaling pathway, which plays a neuroprotective role by inhibiting the activation of NF-κB and subsequent sequence responses (Huang et al., 2021; Gu et al., 2012). The poor hydrophilicity and lipophilicity of ginkgolide B significantly limit its ability to cross the BBB. However, research by Yang Li et al. demonstrated that conjugating ginkgolide B with highly lipophilic docosahexaenoic acid (DHA) to form the covalent complex ginkgolide B-DHA not only enhances the pharmacological effects of ginkgolide B, but also facilitates its stable encapsulation within liposomes. This approach improves solubility and consequently enhances therapeutic efficacy against ischemic stroke (Li et al., 2023).

### 4.5 Alkaloids

Alkaloids with anti-inflammatory activity in ischemic stroke include Ligustrazine and Berberine (Table 5).

**TABLE 5:** Alkaloids inhibit neuroinflammation in ischemic stroke.

Natural plant compound	PubChem CID	Sources	Model	Dose	Mechanisms	References
Ligustrazine (Tetramethylpyrazine)	14296	*Conioselinum anthriscoides* ‘Chuanxiong’ [Apiaceae]	Rats I/R model	40 mg/kg	JAK/STAT signaling pathway	(Qi et al., 2024)
			Rats MCAO model	25, 50 mg/kg	MCPIP1↑ IL-1β, IL-6, TNF-α, MMP-9↓	(Gu et al., 2012)
			Rats MCAO model	20 mg/kg	Circ_0008146-miR-709-Cx3cr1 axis	(Jin et al., 2021)
			Permanent cerebral ischemia rat model	20 mg/kg	Akt and ERK phosphorylation, HMGB1, TLR4, iNOS, NO↓ Nrf2, HO-1↑	(Gong et al., 2019)
Berberine	2353	*Coptis chinensis* Franch. [Ranunculaceae], *Phellodendron amurense* Rupr. [Rutaceae]	OGD/R Rats tMCAO model	10 µM	AMPK signaling pathways	(Mu et al., 2023)
			Rats tMCAO model	25, 50 mg/kg	HMGB1/TLR4/NF-κB signaling	(Ding et al., 2023)
			Rats MCAO/R models	50 mg/kg	IL-1β,TNF-α,IL-6↓ IL-10↑	(Zhu et al., 2019)
			Rats MCAO model	50 mg/kg	Malat1/miR-181c-5p/HMGB1 axis	(Zhu et al., 2018)

I/R, Ischemia reperfusion; MCAO, Middle cerebral artery occlusion; tMCAO, transient middle cerebral artery occlusion; pMCAO, permanent middle cerebral artery occlusion; BBB, blood-brain barrier; ↓, downregulation or inhibition; ↑, upregulation or activation.

#### 4.5.1 Ligustrazine

The traditional Chinese medicine *Conioselinum anthriscoides* ‘Chuanxiong’ [Apiaceae] has the effects of invigorating blood flow and relieving pain, Ligustrazine also known as tetramethylpyrazine, is a potent monomer isolated from the alkaloids of *Conioselinum anthriscoides* because its anti-inflammatory properties are widely used in ischemic stroke (Qi et al., 2024). Mechanistically, tetramethylpyrazine can suppress inflammation and protect BBB integrity by blocking the JAK/STAT signaling pathway as well as upregulating monocyte chemotactic protein-induced protein 1 (MCPIP1) expression (Jin et al., 2021; Gong et al., 2019). The most recent study found that tetramethylpyrazine significantly increased mir-709 expression levels, decreased C-X3-C Motif Chemokine Receptor 1 (Cx3cr1) expression levels, and decreased the concentrations of IL-2 and TNF-α that significantly inhibited cell death, improved neurological scores, reduced infarct volumes. Knocked down circ_0008146 also presented the same effect, suggesting that tetramethylpyrazine targeted circ_0008146/miR-709/Cx3cr1 axis exerts neuroprotective and anti-inflammatory effects (Li L. et al., 2022). Furthermore, tetramethylpyrazine exerts neuroprotective effects in cerebral ischemia by elevating Nrf2/HO-1 expression and inhibiting HMGB1/TLR4, AKT, and Extracellular Signal-Regulated Kinase (ERK) signaling to suppress neutrophil activation, promote endogenous anti-inflammatory defenses, and attenuate proinflammatory responses (Chang et al., 2015). Ligustrazine exhibits low bioavailability and poor BBB permeability; however, nanoparticle and liposome encapsulation strategies demonstrate considerable potential in addressing these limitations (Wen et al., 2022; Mu et al., 2023).

#### 4.5.2 Berberine

Berberine, an isoquinoline derivative alkaloid extracted from medicinal herbs such as *Coptis chinensis* Franch. [Ranunculaceae] and *Phellodendron amurense* Rupr. [Rutaceae], exhibits diverse pharmacological activities (Ding et al., 2023). In ischemic stroke models, berberine demonstrates significant anti-inflammatory effects through multiple molecular mechanisms. The underlying anti-inflammatory mechanisms may involve: (1) modulation of microglial polarization via AMPK signaling pathway regulation (Zhu et al., 2019); (2) suppression of TLR4/NF-κB signaling activation by inhibiting HMGB1 nucleocytoplasmic translocation (Zhu et al., 2018; Duan et al., 2023); and (3) enhancement of butyrate production through gut microbiota modulation to reduce inflammatory cytokine expression. Notably, recent studies have revealed that berberine’s regulatory effect on HMGB1 is associated with lncRNA Malat1, where lncRNA Malat1 competitively binds miR-181c-5p - a direct HMGB1 target - to regulate HMGB1 expression following ischemic stroke (Cao et al., 2020). However, the precise molecular targets directly mediating berberine’s anti-inflammatory actions remain to be fully elucidated. Furthermore, berberine is a substrate of P-glycoprotein and cytochrome P450 enzymes. Its low oral bioavailability and poor BBB penetration significantly limit its clinical application. However, promisingly, numerous studies have demonstrated the potential of novel delivery strategies or encapsulation materials to improve berberine’s bioavailability and BBB permeability (Murakami et al., 2023; Abo El-Enin et al., 2022).

## 5 Discussion and perspectives

Inflammatory storm mediated by activated immune cells and inflammatory mediators constitutes a critical factor exacerbating cerebral tissue damage following ischemic stroke (DeLong et al., 2022). Targeting neuroinflammation has emerged as a pivotal strategy for improving clinical outcomes in stroke patients. Natural plant compounds with diverse bioactivities have been extensively investigated in various disease models, including ischemic stroke. These phytochemicals can attenuate post-ischemic neuroinflammation through multiple mechanisms: suppressing immune cell activation, inhibiting inflammatory mediator production, and blocking peripheral immune cell infiltration. The remarkable anti-inflammatory properties of natural plant compounds position them as promising therapeutic candidates for ischemic stroke treatment (Shang et al., 2022).

Nevertheless, several unresolved challenges persist in advancing natural plant compounds from preclinical research to clinical applications for stroke therapy. The primary limitation lies in their suboptimal bioavailability. Many phytochemicals exhibit poor water solubility, directly compromising their absorption and distribution. Furthermore, the selective permeability of the BBB substantially restricts compound delivery to cerebral ischemic regions (Huang et al., 2024). A second critical issue involves ambiguous pharmacological targets and mechanisms of action. While numerous natural products demonstrate anti-inflammatory properties in experimental stroke models, their precise molecular targets and downstream signaling pathways remain incompletely characterized. This knowledge gap hinders rational drug optimization and target validation. Thirdly, methodological limitations in preclinical studies warrant attention: Translational relevance is compromised by predominant use of young animals in modeling a predominantly geriatric disease (Finger et al., 2022); Temporal dynamics of therapeutic evaluation require refinement, as most investigations focus on acute phase outcomes despite the chronic nature of post-stroke inflammation (Ahsan et al., 2021); Standardization issues persist regarding dosage regimens (administration routes and therapeutic windows), introducing variability that undermines inter-study comparability. Finally, clinical translation faces dual barriers: The translational gap between rodent models and human pathophysiology, particularly regarding comorbidities and aging-related biological changes; Significant inter-patient heterogeneity in stroke manifestations and treatment responses, necessitating large-scale clinical trials to ensure robust conclusions. Addressing these challenges through integrated pharmacokinetic optimization, mechanism-driven research, standardized preclinical protocols, and rigorous clinical validation will be crucial for harnessing the therapeutic potential of natural plant compounds in stroke management (Tao et al., 2020; Gong et al., 2023).

Recent advancements in pharmacology, molecular biology, nanotechnology, and artificial intelligence have unveiled novel therapeutic avenues for natural plant compounds in stroke management (Tao et al., 2020). Notably, nanoparticle-based delivery systems have demonstrated significant potential in overcoming solubility limitations and enhancing BBB penetration. For instance, curcumin-loaded nanoparticles effectively improve bioavailability and facilitate anti-inflammatory effects in cerebral ischemia models (Li F. et al., 2021). Additionally, baicalin nanoparticles delivered via the intranasal route successfully target brain tissues in experimental stroke models (Yu et al., 2023). The integration of omics technologies, including transcriptomics, metabolomics, and proteomics, has substantially accelerated mechanistic investigations of various natural plant compounds in ischemic stroke. Concurrently, artificial intelligence-assisted molecular docking techniques have been widely adopted for natural target screening, proving crucial for identifying direct molecular targets and elucidating pharmacological mechanisms of natural plant compounds. Moreover, while clinical trials directly investigating natural plant compounds in ischemic stroke remain limited, their safety profiles have been extensively documented through clinical studies in other disease contexts (Bai et al., 2025; Conforti et al., 2024). This existing evidence provides a foundation for conducting well-designed clinical trials under strict ethical guidelines to evaluate natural product-based interventions for ischemic stroke. Such systematic clinical investigations would significantly advance the clinical translation of natural therapeutics for cerebrovascular protection, potentially offering novel treatment strategies to improve neurological outcomes in stroke patients.
